# Optimal Sensor Selection for Classifying a Set of Ginsengs Using Metal-Oxide Sensors

**DOI:** 10.3390/s150716027

**Published:** 2015-07-03

**Authors:** Jiacheng Miao, Tinglin Zhang, You Wang, Guang Li

**Affiliations:** State Key Laboratory of Industrial Control Technology, Institute of Cyber Systems and Control, Zhejiang University, Hangzhou 310027, Zhejiang, China; E-Mails: jiacheng@zju.edu.cn (J.C.M.); ztlyoyo@163.com (T.L.Z.); guangli@zju.edu.cn (G.L.)

**Keywords:** sensor selection, metal-oxide sensors, classification, electronic nose, linear discriminant analysis

## Abstract

The sensor selection problem was investigated for the application of classification of a set of ginsengs using a metal-oxide sensor-based homemade electronic nose with linear discriminant analysis. Samples (315) were measured for nine kinds of ginsengs using 12 sensors. We investigated the classification performances of combinations of 12 sensors for the overall discrimination of combinations of nine ginsengs. The minimum numbers of sensors for discriminating each sample set to obtain an optimal classification performance were defined. The relation of the minimum numbers of sensors with number of samples in the sample set was revealed. The results showed that as the number of samples increased, the average minimum number of sensors increased, while the increment decreased gradually and the average optimal classification rate decreased gradually. Moreover, a new approach of sensor selection was proposed to estimate and compare the effective information capacity of each sensor.

## 1. Introduction

Artificial olfaction systems, or electronic noses (E-noses) are devices designed for mimicking the mammalian sensory system and they have been applied for odor assessment in the field of environmental quality monitoring [1,2], food and beverage quality control [3,4,5,6], medical diagnosis [7,8,9] and others. Sensor selection is one of most important issues [10,11,12,13] in the artificial olfaction system field. The goal of sensor selection is to optimize the sensor subset [14,15,16,17,18,19,20]. The feature/sensor selection techniques are grouped into two categories: Filters and wrappers. In filter methods, sensors are selected on the basis of the information content provided by a combination of sensors. The information content only takes into account the relationships of sensors. In wrapper methods, sensors are selected by the predictive accuracy of a model trained on the particular sensor subset. Each approach has advantages and disadvantages, and the relationship between these two approaches is vague. However, in general, wrapper methods lead to better results than filter methods [21].

The target optimal sensor array should be sensitive to the target odors and each sensor should provide different information about the odors. When constructing a sensor array, the optimization of the number of the sensors is essential, since excessive sensors often import redundant, uncorrelated information, or even noise, which negatively affects the performance of the learning algorithm. In addition, the total feature for one measurement is the product of number of sensors and number of features extracted by each sensor, which usually consist of steady-state values and transient dynamic responses. Therefore, an excessive number of sensors will lead to the problem known in the machine learning field as curse of high dimensionality. 

In the present work, we systematically investigated the optimal sensor selection problem by testing ginsengs. *Panax ginseng* C.A. Meyer is mainly cultivated in Korea and northeast China, and American ginseng (*Panax quinquefolius*) is mainly cultivated in North America and China (they are called by the shared name ‘ginseng’ in this paper). Ginsengs are widely used in Chinese Traditional Medicine. The major biologically effective constituents of ginsengs are the ginsenosides. Besides, ginsengs also contain polysaccharides, proteins, amino acids, volatile oils and other components. Ginseng volatile oils are mainly constituted of sesquiterpenoids, long chain saturated carboxylic acids and some aromatic hydrocarbons [22,23]. The constituents of ginsengs differ between species, production places and processing techniques. 

In most E-nose application cases, as many as available sensors were used without estimating the number of sensors needed for the case. Though some researchers have applied sensor selection techniques to reduce the number of sensors used in the models during the data processing [14,15,16,17], the importance of each sensor was not estimated and how well each sensor ‘cooperates’ with others was unclear. These approaches are uneconomic and not environmentally friendly. In the meanwhile, as the number of sample categories increases, how the minimum number of sensors needs to change and how the classification performance changes are still unclear. 

In this paper, firstly, the minimum number of sensors needed for discriminating a certain sample set was defined and investigated with an exhaustive method by classifying the sample set with all the potential sensor sets (at least one sensor included) using linear discriminant analysis (LDA). Then, the minimum number of sensors needed for the sample set with same number of sample categories was demonstrated by investigating the corresponding minimum number of sensors for all the potential sample sets (at least two samples included). Finally the relation between the minimum number of sensors and the corresponding classification performance and the number of sample categories within a sample set was revealed and discussed. Moreover, a new sensor selection/estimation approach has been developed by comparing the average classification performance of sample sets including certain sensor with those not included. The impact of sensor failure on system robustness was also discussed.

## 2. Experimental Section

### 2.1. Sample Preparation

The ginseng samples (Table 1) were randomly purchased from the Changchun Medicinal Material Market (Changchun, China). The ginseng samples were pulverized and 10 g was placed in 100 mL headspace vial, sealed and placed in a thermostat at 50 °C for 30 min. Then 10 mL headspace gas was extracted with a syringe for one measurement.

**Table 1:** Details of the samples.

Sample No.	Ginseng Samples	Places of Production
1	Chinese red ginseng	Ji’an
2	Chinese red ginseng	Fusong
3	Korean red ginseng	Ji’an
4	Chinese white ginseng	Ji’an
5	Chinese white ginseng	Fusong
6	American ginseng	Fusong
7	American ginseng	USA
8	American ginseng	Canada
9	American ginseng	Tonghua

### 2.2. E-Nose Equipment

**Figure 1:**
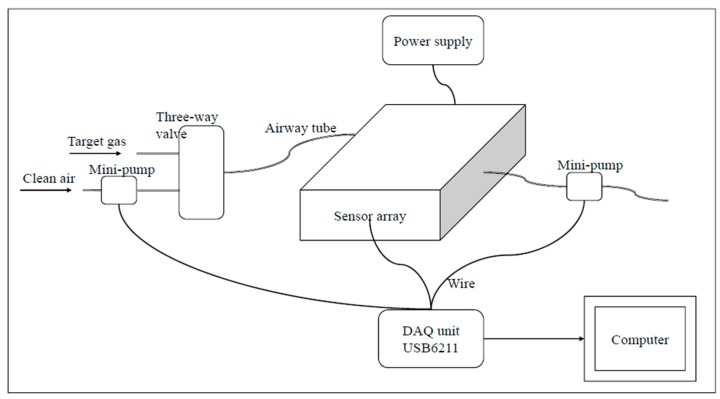
The schematic diagram of the E-nose system.

The schematic diagram of the homemade E-nose system used is shown in Figure 1. Twelve metal oxide sensors were purchased from Figaro Engineering Inc. (Osaka, Japan) and fixed on a printed circuit board, which was placed in a 200 mL stainless chamber. The response characteristics of the sensors are shown in Table 2. A three-way valve is used to switch between target gas and clean dry air. Two mini vacuum pumps are used for gas washing at a constant flow of 1 L/min and controlled by the computer. A USB6211data acquisition (DAQ) unit, purchased from National Instruments Inc. (Austin, TX, USA), is used to acquire the sensor signals and control the pumps.

The heating temperature of a metal-oxide semi-conductive sensor is very important, and variance in the applied heating temperature will cause the sensor’s characteristics to change and more valid information may be obtained [24]. To make our problem simpler, we set the heater voltage of each sensor at 5 V DC, which is recommended by Figaro Inc. to maintain sensor’s characteristics at a fixed heating temperature.

**Table 2:** Response characteristics of the sensors.

No.	Sensor Type	Response Characteristics
1	TGS813	Carbon monoxide, ethanol, methane, hydrogen, isobutane
2	TGS821	Carbon monoxide, ethanol, methane, hydrogen
3	TGS822	Carbon monoxide, ethanol, methane, acetone, n-Hexane, benzene, isobutane
4	TGS822	Carbon monoxide, ethanol, methane, acetone, n-Hexane, benzene, isobutane
5	TGS826	Ammonia, trimethyl amine
6	TGS832	R-134a, R-12 and R-22, ethanol
7	TGS800	Carbon monoxide, ethanol, methane, hydrogen, ammonia
8	TGS880	Carbon monoxide, ethanol, methane, hydrogen, isobutane
9	TGS2600	Carbon monoxide, hydrogen
10	TGS2602	Hydrogen, ammonia ethanol, hydrogen sulfide, toluene
11	TGS2610	Ethanol, hydrogen, methane, isobutane/Propane
12	TGS2611	Ethanol, hydrogen, isobutane, methane

### 2.3. Measurement

**Figure 2:**
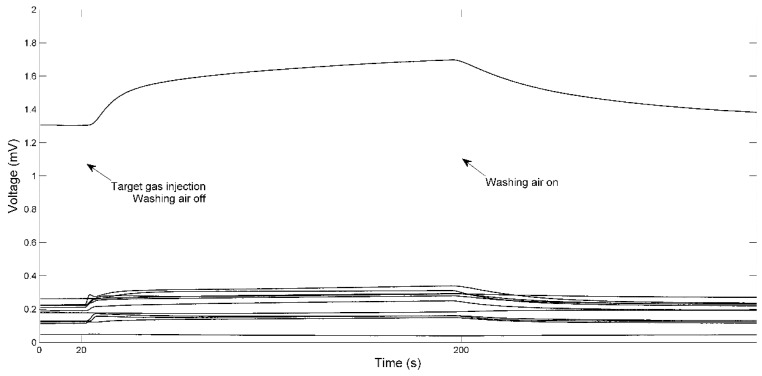
Example of responses of 12 sensors to ginseng sample.

The gas chamber was washed with a clean-air flow of 1 L/min for 360 s to allow the sensors to return to the baseline before another new cycle of measurements. Then the clean-air flow was turned off and the target gas was quickly injected into the chamber with a syringe. The response of sensors was collected for 180 s, and then the clean-air flow was turned on to wash away the target gas. The response of 12 sensors was recorded for 340 s at 2 Hz during each testing, including 20 s before and 140 s after the measurement response of sensors, as shown in Figure 2. A total of 315 samples, 35 for each species, were obtained. The experiment was conducted at room temperature (20–25 °C) and humidity of 50%–70%. During one measurement (which usually takes 10–15 min), the temperature and humidity of ambient environment were relatively stable.

### 2.4. Data processing

The response of each sensor is calibrated by:

$$R=\left(R_{s}-R_{o}\right)/R_{o}$$

where *R_s_* is the actual signal of sensor, *R_o_* is the baseline value. The calibration procedure can partly compensate for any sensor drift [16,25,26]. To simplify our problem, features at the same time points for each sensor were extracted. To define a reasonable set of features, a series of value of *R* were firstly extracted as the candidate features at the time points of 25, 30, 40, 50, 60, 70, 120, 170, 220, 270 and 320 s. Then an exhaustive search algorithm was used to find the optimal subset of candidate features to classify the whole nine classes of samples. The result showed that the best classification result was achieved with feature set of time points that consisted of 25, 30, 60, 70 and 170 s. Therefore, five features at those time points were extracted for each sensor. 

The Linear Discriminant Analysis (LDA) algorithm, Support Vector Machine (SVM) and *k*-nearest Neighbor (KNN) with *k* = 1,3,5,7 were compared and the most appropriate classification algorithm was employed in Section 3.2, Section 3.3 and Section 3.4 based on the classification accuracy and computational complexity of the classification algorithm. SVM was performed with the libsvm [27] toolbox using C-SVC (SVM classification with cost parameter C) with nonlinear kernel of Radial Basis Function (RBF). C was set to be 104 and *γ* (parameter of kernel RBF) was searched with log 2 (*γ*) = − 5, −4, …, 5.

All the classification results in this paper have been applied with 10-fold cross-validation: The dataset was divided into training set and testing set. For each ginseng sample, 35 measurements were randomly divided into 10 sets, with three or four measurements in one set. Leave-one-out validation was repeated 10 times until every set had been used as a testing set. Finally, the entire procedure was repeated 10 times. So every classification result reported below is the average performance of training and testing 100 classifiers.

## 3. Results and Discussion

We investigated the overall performance of all potential sensor sets with *N* (*N* = 1 to 12) sensors for classifying all potential sample sets with *M* (*M* = 2 to 9) ginsengs. Therefore, we got 2^9^ – 9 – 1 = 520 different sample sets to discriminate with 2^12^ – 1 = 4095 different sensor sets. For example, if we want to discriminate a sample set consisting of ginsengs Nos. 1, 3, 4 and 6 (see sample details in Table 1) with a sensor set consisting of sensors Nos. 2, 4, 5, 7 and 11 (Table 2), the dimensions of the dataset for training and testing the classifier is 140 × 25 (4 × 35 measurements with 5 × 5 features).

### 3.1. Comparison of Different Classification Algorithms

**Figure 3:**
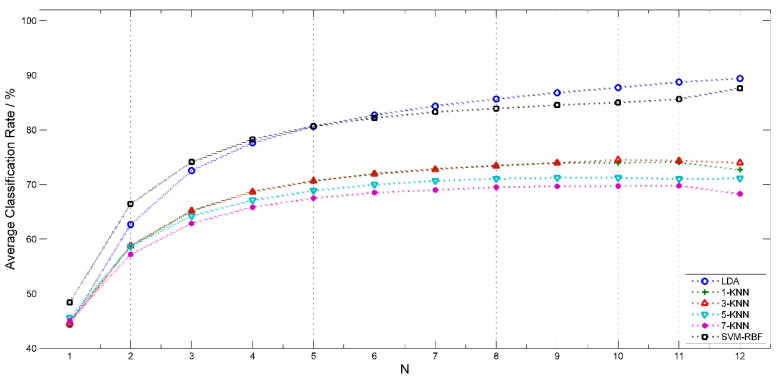
Comparison of average classification accuracy of sensor sets with *N* (*N* = 1 to 12) sensors for LDA, SVM and KNN (*k* = 1, 3, 5, 7).

LDA, SVM and KNN (*k* = 1, 3, 5, 7) were employed and compared for discriminating sample set *A1* = 1, 2, 3, 4, 5, 6, 7, 8, 9 (with all nine classes of sample) with all potential sensor sets. The average classification accuracy of sensor sets with *N* (*N* = 1 to 12) sensors were compared and shown in Figure 3. LDA and SVM-RBF achieved better classification accuracy than all KNNs except for LDA with *N* = 1. The performance of LDA and SVM-RBF is near, SVM-RBF performs better with *N* < 5 and LDA performs better with *N* > 5. Total time consumption of LDA and SVM-RBF is 1113 s and 47,599 s separately. SVM-RBF takes much more time than LDA. Thus, based on the assessment of classification accuracy and computational efficiency, LDA was chosen and employed in the following analysis, except for Section 3.5.

### 3.2. Classification Performance of All Potential Sensor Sets for All Potential Sample Sets

**Figure 4:**
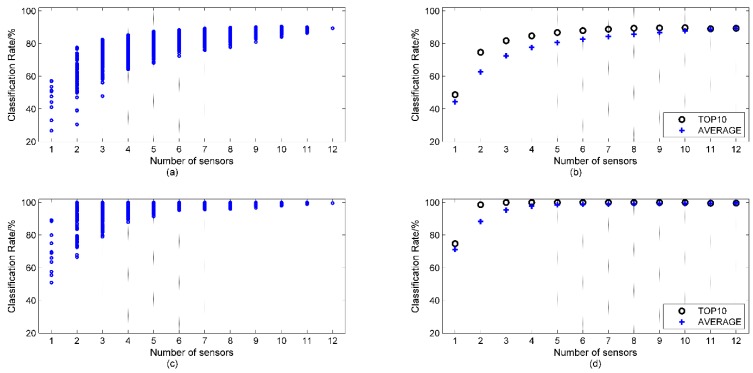
(**a**) Classification performance of sample set *A1* with *N* (1 to 12) sensors; (**b**) Corresponding TOP 10 and AVERAGE value; (**c**) Classification performance of sample set *A2* with *N* (1 to 12) sensors; (**d**) Corresponding TOP 10 and AVERAGE value.

For a certain sample set *A*，the average top 10 classification performance of sample sets with *N* sensors represents the optimal result with sensor set of *N* sensors. As *N* increases, the maximum ‘optimal result’ will be obtained with a certain value of *N* and this value defines the minimum number of sensors needed for discriminating sample set *A*. Hence, firstly, the overall classification performance of 4095 sensor sets for 502 sample sets were calculated. Due to limited space, the classification performances of sample set *A1* = 1, 2, 3, 4, 5, 6, 7, 8, 9 with all nine classes of sample and *A2* = 2, 5, 9 with Nos. 2, 5, 9 samples are shown as typical examples in Figure 4a,c. *A1* is the most complex classification target in our experiment; *A2* was randomly selected with fewer kinds of samples and considered to be less complex compared to *A1*. For a certain sample set, there are ${\text{C}}_{12}^{\text{N}}$ classification performances with *N* sensor sets (*N* = 1 to 12) sensors. The average of the top 10 classification performances (TOP 10) and the average of all classification performances (AVERAGE) with *N* sensors are shown in Figure 4b,d for sample set *A1* and *A2* (when *N* = 12, TOP 10 = AVERAGE, for there is only one sensor set of 12 sensors). The number of sensors where TOP 10 achieves a maximum value is defined as the minimum number *N_min_* (*A*) for the discrimination of sample set *A*, and if TOP 10 has more than one maximum value, the minimum *N* with maximum value is taken as *N_min_* (*A*). 

For sample set *A1*, the classification rates of individual sensors (see Figure 4a, *N* = 1) range from 19% (No. 2 sensor) to 57% (No. 9 sensor), which indicates the discrimination ability of individual sensors. The width of the range decreases as the number of sensor (*N*) increases. The AVERAGE value increases with *N*. However, the TOP 10 reaches the maximum value with *N* equal to 10, which means the most appropriate number of sensors for discriminating sample set *A1* is 10, *i.e.*, *N_min_* (*A*) = 10. For sample set *A2*, many sensor sets’ classification performance reach 100% with two to four sensors, because discriminating sample set *A2* is an easier problem compared to *A1*. The TOP 10 values are all 100% with *N* = 3 to 9. According to definition of *N_min_* (*A*) above, *N_min_* (*A2*) = 3.

### 3.3. An Approach for Grading the Sensors for the Discrimination of a Certain Sample Set

**Figure 5:**
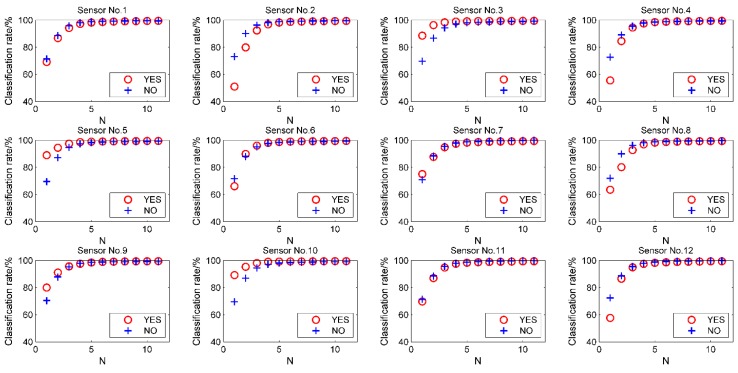
The first step of comparison of average classification performance (*A2*) of sensor sets including certain sensor with that not including it for sensor number of *N* = 1 to 11. ‘YES’ means including, ‘NO’ means not including.

When a certain sensor set is used to discriminate a certain sample set, some sensors may contain more valid information, while some may contain less valid or redundant information. Some sensors even carry a lot of noise that degrades the performance of the classifier. In this section, we come up with a new approach to grade the sensors within the sensor set for discriminating a certain sample set. Firstly, for a certain sample set consisting of *M* sample species, the classification accuracies of each potential combination of sensors were calculated. We compared the average performance of sensor sets of *N* (*N* = 1 to 11) sensors including certain sensors with those not including it. ‘+’ was added after sensor’s serial number for ‘better when including it’, and ‘−’ for ‘worse when including it’. Then we deleted the sensor sets including sensors with ‘−’ values. The entire procedure was repeated until no sensor was added a ‘−’. Finally, the sensors are graded according to performance during the whole procedure. This procedure does not need any initial condition or parameter, and the selection result in every step is fixed after calculating the classification accuracy of all combinations of sensors. Taking sample set *A2* = 2, 5, 9 as an example, the result of the first step of the procedure was shown in Figure 5 and the results are listed in Table 3. 

We can see that sensors Nos. 3 and 10 always have a ‘+’ during the whole procedure, from which can deduce that they may contain more effective information than the others, and ‘cooperate’ well with other sensors for the discrimination of sample set *A2*. Nos. 2, 8, 11, 12 sensors are deleted in the second step. However, it doesn’t mean they are useless. They just contain less useful information or more noise than sensors Nos. 3 and 10, or some information is redundant. Some of them still help make the result better, because the best classification rate for sample set *A2* (100%) comes with sensor sets containing one or more of them. No. 9 sensor doesn’t show up with ‘+’ or ‘−’ in the table, which mean its performance is better than sensors with ‘−’ and worse than those with ‘+’. Then we concluded that Nos. 3 and 10 sensors are in the first grade sensors containing the most effective information, Nos. 5 and 9 are in the 2nd grade, Nos. 4 and 6 are in the 3rd grade, Nos. 1 and 7 are in the 4th grade and the rest are in 5th grade.

**Table 3:** Sensor grading procedure.

Step	Procedure	Sensors Estimation
1	Start	No. 3 +, 5 +, 10 +, 2 −, 8 −,11 −,12 −
2	Deleting No. 2, 8, 11, 12 sensors	No. 3 +, 10 +, 1 −, 7 −
3	Deleting No. 1, 7 sensors	No. 3 +, 10 +, 4 −, 6 −
4	Deleting No. 4, 6 sensors	No. 3 +, 10 +
5	Stop	

### 3.4. The Minimum Number of Sensors for All Potential Sample Sets

**Figure 6:**
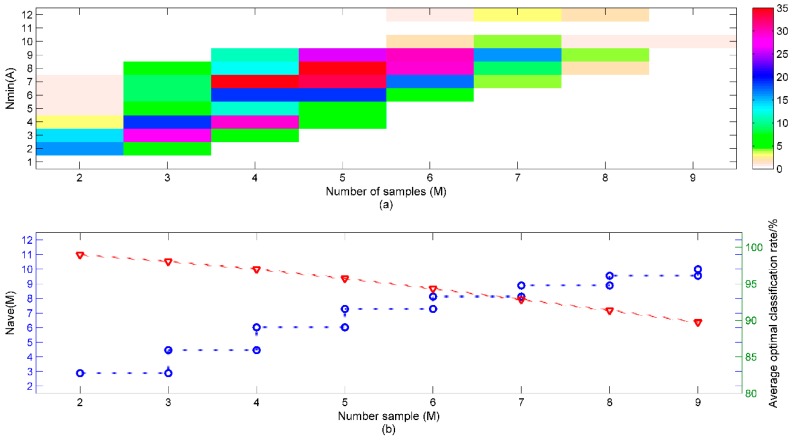
(**a**) Distribution of the sample sets with *M* samples, color bar indicates the number of the sample; (**b**) The average *N_ave_* (*A*) (circle) of sample sets with *M* samples and corresponding optimal classification performances (triangle).

The minimum numbers of sensor *N_min_ (A)* and corresponding average classification performances for all potential sample sets were obtained with the method mentioned in Section 3.2 and shown in Figure 6a. For different sample sets with the same number of samples, *N_min_* (*A*) varies a lot, since the complexity for discriminating different sample sets is different. By setting different sample sets with different numbers of sample species, we wanted to investigate how the minimum number of sensors varies as the number of sample species increases. When we averaged *N_min_ (A*) and the corresponding classification performances, the result is shown in Figure 6b. It is observed that as the number of samples (*M*) increases, the average minimum number of sensors *N_ave_* (*M*) also increases, whereas the average classification accuracy decreases smoothly. For example, when *M* = 2, *N_ave_* (2) is 2.9, and corresponding average optimal classification rate is 99.0%, and when *M* = 3, *N_ave_* (3) is 4.5, and corresponding average optimal classification rate is 99.1%. We also noted that the increment of *N_ave_* (*M*) decreases as *M* increases. The increment of *N_ave_* (*M*) is about 1.6 when *M* grows from 2 to 3, whereas it is just about 0.4 when *M* grows for 8 to 9. We deduced that when we gradually add more ginseng samples to the existing nine ginsengs, *N_ave_* (*M*) will gradually to be approximately constant if *N_ave_* (*M*) doesn’t reach 12, and the average optimal classification performance of sample sets with *M* samples will still decrease gradually. Because when *M* increases, the classification problem will become more complex, but all existing sensors would not provide sufficient discriminant information, then *N_ave_* (*M*) gradually will become approximately constant while the classification performance gradually decreases. Furthermore, we deduced that if there were a large set of samples and sufficient but not excessive sensors, when the number of samples to discriminate increased, the minimum number of sensors would increase and gradually verge to constant while the optimal classification performance decreased gradually.

### 3.5. Impact on System Robustness by Sensor Failure

Sensor failure means an alteration of characteristic response either caused by the nature of the sensor itself (aging or poisoning) or by degraded measurement conditions (deteriorated electrical contacts, degraded data condition circuitry or a stressed sensor). The altered response may be low, uneven or intermittent [26]. We assumed a flat response was acquired for a failed sensor and the five features corresponding to a failed sensor was set to 0 for testing samples, as proposed in [26].

**Figure 7:**
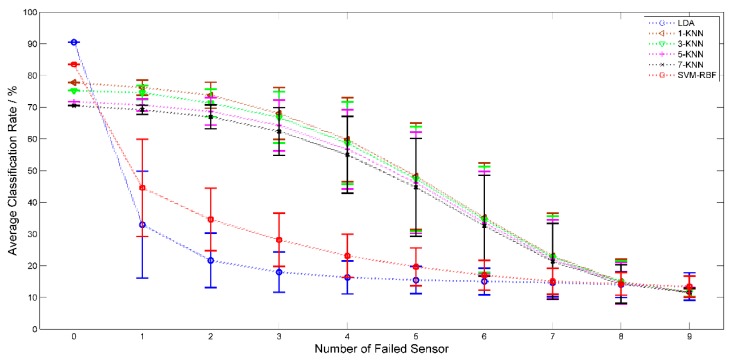
The mean standard deviation of classification accuracy with increasing number of failed sensor for LDA, SVM-RBF and KNN (*k* = 1, 3, 5, 7).

The optimal classification accuracy of sample set *A1* = 1, 2, 3, 4, 5, 6, 7, 8, 9 was achieved with 10 (out of 12) sensors in Section 3.2. System robustness for sample set *A1*was investigated with *P* (*P* = 0, 1… 9) (out of 10) failed sensors. For *P* failed sensors, there were $C_{10}^{p}$ possible failure combinations. The mean and standard deviation of the system classification accuracy with *P* failed sensors were used to evaluate system robustness. LDA, SVM-RBF and KNN (*k* = 1, 3, 5, 7) were employed and compared as classifier. From Figure 7, we observed that performance of LDA and SVM-RBF is critically affected by sensor failure while KNN performs much better than the other two classifiers. How to improve the robustness of a system suffering from sensor failure is a meaningful subject to which we will pay attention in future work.

## 4. Conclusions

In the present work, we measured nine ginsengs with a homemade E-nose system consisting of 12 metal oxide sensors. We investigated the classification performance of 4095 sensor sets for 502 sample sets. The minimum number of sensors for all sample sets was defined and calculated. We found that as the number of samples increases, the minimum number of sensors needed increases while the increment gradually decreases. In the meanwhile, the average classification performances decrease gradually. Our research provides instructive advice on choosing an appropriate number of sensors for the target in E-nose applications, even though the relationship between the minimum number of sensors and the number of samples may not be exactly the same when dealing with different target samples. We also came up a new approach to grade sensors by comparing the average classification performance of sample sets including certain sensors with those not.

In the future, we plan to investigate why certain combination of sensors performs better than others for certain combination of samples, and how can we use this information to improve the sensor estimation procedure. How to improve the robustness of a system suffering from sensor failure is also a meaningful subject that we will pay attention to in future work.
